# Needleless administration of advanced therapies into the skin via the appendages using a hypobaric patch

**DOI:** 10.1073/pnas.2120340119

**Published:** 2022-04-28

**Authors:** Faiza Benaouda, Ricardo Inacio, Chui Hua Lim, Haeeun Park, Thomas Pitcher, Mohamed A. Alhnan, Mazen M. S. Aly, Khuloud T Al-Jamal, Ka-lung Chan, Rikhav P. Gala, Daniel Sebastia-Saez, Liang Cui, Tao Chen, Julie Keeble, Stuart A. Jones

**Affiliations:** ^a^Institute of Pharmaceutical Science, Faculty of Life Sciences & Medicine, King’s College London, London SE19NH, United Kingdom;; ^b^Basic & Medical Biosciences, Faculty of Life Sciences & Medicine, King’s College London, London SE1 1UL, United Kingdom;; ^c^Fraunhofer USA Center for Molecular Biotechnology, Newark, DE 19711;; ^d^Department of Chemical and Process Engineering, University of Surrey, Surrey GU2 7XH, United Kingdom;; ^e^Department of Civil and Environmental Engineering, University of Surrey, Surrey GU2 7XH, United Kingdom;; ^f^Centre For Pharmaceutical Medicine Research, Faculty of Life Sciences & Medicine, King's College London, London SE19NH, United Kingdom

**Keywords:** needleless delivery, advanced therapeutics, skin, drug delivery, vaccine

## Abstract

Advanced therapies are commonly administered via injection even when they act within the skin tissue, and this increases the chances of off-target effects. Here we report the use of a skin patch containing a hypobaric chamber that induces skin dome formation to enable needleless delivery of advanced therapies directly into porcine, rat, and mouse skin. Finite element method modeling showed that the hypobaric chamber in the patch opened the skin appendages by 32%, thinned the skin, and compressed the appendage wall epithelia. These changes allowed direct delivery of an H1N1 vaccine antigen and a diclofenac nanotherapeutic into the skin. Fluorescence imaging and infrared mapping of the skin showed needleless delivery via the appendages. The in vivo utility of the patch was demonstrated by a superior immunoglobulin G response to the vaccine antigen in mice compared to intramuscular injection and a 70% reduction in rat paw swelling in vivo over 5 h with diclofenac without skin histology changes.

## Significance

Needleless delivery into the skin would overcome a major barrier to efficient clinical utilization of advanced therapies such as nanomaterials and macromolecules. This study demonstrates that controlled skin stretching (in porcine, rat, and mouse models) using a patch comprising a hypobaric chamber, to open the skin appendages, can increase the permeability of the tissue and provide a means to enable direct delivery of advanced therapies directly into the skin without the use of a needle or injection system. This technology can facilitate the self-administration of therapeutics including vaccines, RNA, and antigens, thus improving the translation of these products into effective clinical use.

The skin viable epidermis is an important target site for both locally acting therapeutics and immunotherapies. However, this layer is sandwiched between the outer, lipidic stratum corneum (SC) and the underlying, blood-rich dermis, and thus it is very difficult to access. Hydrophobic agents that are applied to the surface of the skin have a good affinity with the SC, but they have difficulty passing out of this layer into the underlying hydrophilic epidermis. In contrast, hydrophilic agents have difficulties penetrating the SC due to a lack of affinity with the tissue’s lipids and its very small pores (1). Agents administered via injection into the blood are diluted in the circulation and thus produce a low local tissue concentration. The difficulties encountered when trying to access the epidermis diminishes the clinical impact of numerous frequently used medicines including locally delivered corticosteroids (2), nonsteroidal antiinflammatory agents (3), topical cancer chemotherapy (4), and agents used to treat superficial manifestations of viral infections (5). It also makes it very difficult to administer advanced therapies such as tissue regenerative therapy and immunotherapies that can stimulate effective responses if they reside in the skin (6).

Some of the most exciting new therapeutic agents that are in development for skin diseases take the form of macromolecules or nanomaterials. These large molecules (typically with a size of >10 nm) can localize in the skin epidermis if they can be delivered through the SC because the basement membrane, a region that separates the epidermis from the dermis, slows drug clearance from the epidermis into the dermal tissue, thus to the systemic circulation (7). For example, liposomes can localize hydrocortisone in the epidermis and limit clearance into the systemic circulation (8). However, to date, this effect has only been observed when the SC is removed before drug administration using tape stripping because liposomes, like nanostructured lipid carriers (9), niosome (10), and dendrimers (11), have difficulty in passing through the SC after topical application to the skin.

Forming micropores in the skin can increase the permeability of the SC to large therapeutic agents. This can be achieved temporarily and noninvasively by applying mechanical pressure to the skin (12). However, a micropore formation approach that can be rapidly reversed, to limit the ingress of foreign material, has yet to be developed to meet the need of chronic skin conditions, which require repeatable daily drug administration.

The stretching of the skin that occurs during massage has been shown to open the skin appendages and enhance the delivery of agents into the skin after topical application (13, 14). Skin stretching has the advantage over micropore formation, when attempting to administer therapeutic agents into the skin, in that it is noninvasive but also rapidly reversed by the energy stored in the skin collagen, which relaxes the tissue after the application of tissue strain (15). In addition, skin stretching can open hair follicles blocked with sebum to allow the passage of agents into the skin after topical administration (16–18). However, skin massage does not impart a controlled skin stretching and there is a need to develop a simple low-cost device that can reproducibly stretch the skin and facilitate drug delivery directly into the tissue.

Suction devices that stretch the skin using hypobaric pressure have been developed to improve burn therapy (19), wound healing (20), skin laser therapy (21), and the in vivo transfection of DNA after intradermal injection (22). The safety of skin stretching using suction has been well-established using these devices and it has been extensively studied as a means to determine skin elasticity; for example, the Cutometer, a commercial hypobaric system that uses 500 mbar of pressure to measure skin elasticity in humans, has been reported in >200 studies. The literature published on the in vivo use of the Cutometer has shown that the device parameters drive how hypobaric pressure modifies the skin; for example, the device aperture size influences the depth of tissue deformation (23). However, at present, there are no commercially available devices that stretch the skin to allow the direct delivery of therapeutic agents into the tissue.

**Fig. 1.**
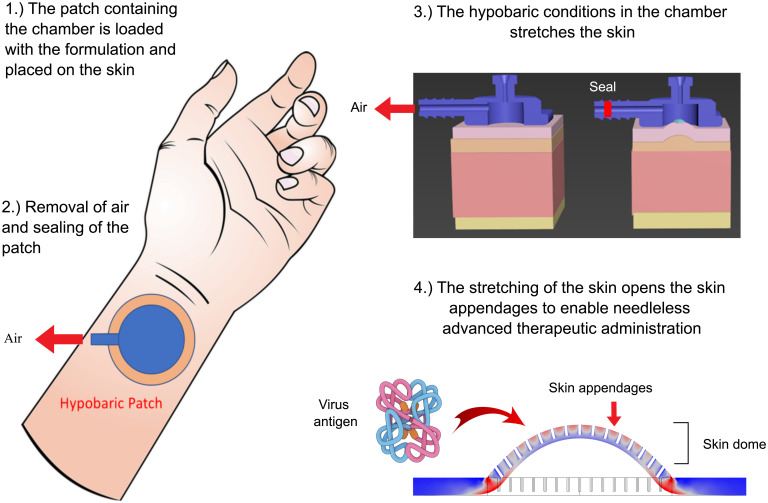
A schematic showing the proposed clinical use of the patch with the hypobaric chamber loaded with the drug-containing formulation and applied to the skin.

In this work, we demonstrate that applying a patch containing a hypobaric drug delivery chamber can generate controlled tissue stretching that enhances the direct delivery of macromolecules and nanomaterials into porcine and rodent skin through depressurization that can be achieved using a vacuum. We follow the distribution of the administered materials in the tissue and show that they pass into the skin through appendages that were opened by the hypobaric chamber in the patch. We demonstrate the in vivo viability of the patch using a rat inflamed paw model and through the efficacy of an H1N1 HA antigen vaccine. This innovative patch can be cost-effectively mass-produced, it is needle- and pain-free, and it can be attached to a vacuum tube, syringe, or vacuum box (the size of a heart monitor) for 5 s to actuate the hypobaric pressure, which initiates drug administration; thus, it is an attractive technology for future clinical application in humans (Fig. 1).

## Results

### The Hypobaric Chamber in the Patch Induced Skin Dome Formation That Opened the Appendages.

**Fig. 2.**
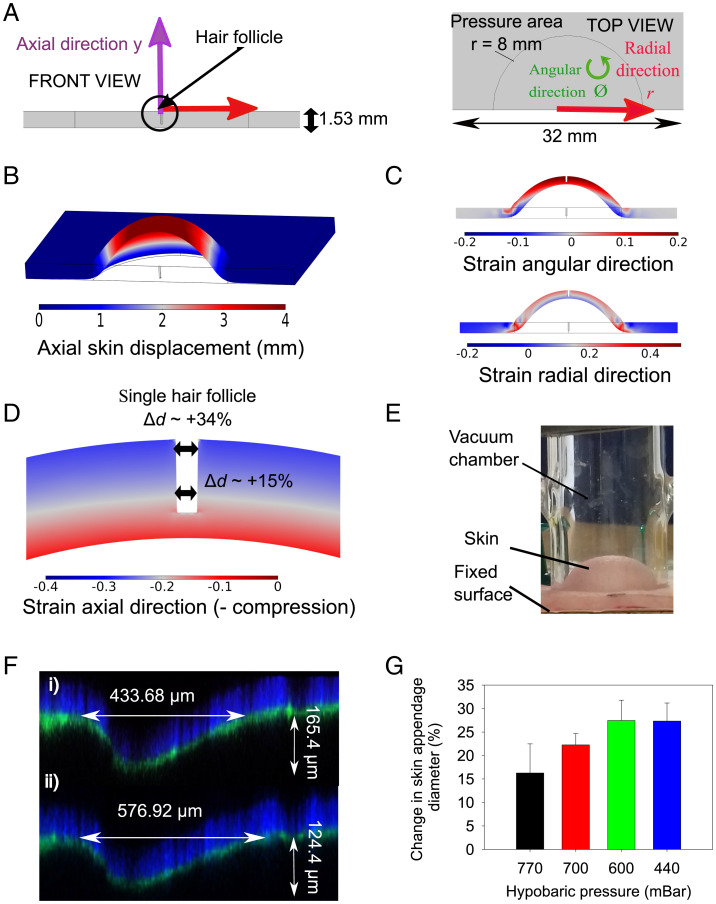
(*A*) The FEM model geometry. (*B*) A three-dimensional FEM model image of application of 500 mbar using the hypobaric chamber. (*C*) A FEM cross-sectional image of angular and radial strain components. (*D*) A FEM visualization of the hair follicle deformation during hypobaric chamber application. (*E*) A photograph of the skin dome formed on porcine skin ex vivo using a hypobaric chamber at 500 mbar. (*F*) Confocal microscopic images of the skin appendages (*i*) without hypobaric pressure and (*ii*) with hypobaric pressure. (*G*) The mean relative difference in diameter of the skin appendages at different hypobaric conditions compared to atmospheric pressure (asterisk indicates significantly different from atmospheric pressure of ≈1,018 mbar). Data in *G* are ± SEM (*n* = 3).

The depressurization of the chamber in the patch generated a controllable hypobaric pressure when applied to the surface of the skin that could be measured using a digital manometer. This process was modeled using finite element method (FEM) modeling to understand the stresses applied to the skin and the resultant tissue deformation. The model predicted that applying hypobaric conditions to the chamber in the patch, which had a circular aperture that sealed on the skin surface, resulted in skin dome formation with strain in the axial (y), radial (r), and angular directions (ϕ) (Fig. 2 *A–D*). The application of the microchamber caused the most significant skin displacement in the axial direction (Fig. 2*B*). It was also notable that the overall skin thickness decreased by 13 to 20% across the tissue. The radial and angular skin stretch decreased from the top skin surface to the bottom surface and from the center to the edge (Fig. 2*C*). Inserting a skin appendage, with dimensions that mimicked in vivo morphometric data of a hair follicle, indicated that the appendage epidermis was compressed (generating negative strain values), especially toward the outer surface of the skin (Fig. 2*D*). The model predicted that using 500 mbar of pressure would cause the appendage to open by 34% at the surface and 15% in the lower regions of the dermis when positioned in the middle of the dome. Inserting appendages at different places across the skin dome gave an average appendage opening of 32 ± 3%.

To verify the FEM modeling data, we assessed the nature of the skin deformation upon the application of a glass hypobaric chamber visually (Fig. 2*E*). A skin dome was observed, and the skin appendages were visibly larger when the chamber was applied to the tissue, so we then used a StageFlexer hypobaric cell to quantify the extent of skin appendage opening using confocal microscopy (*SI Appendix*, Figs. S1 and S2). At 440 mbar the average increase in the appendage width across the skin dome was 27 ± 7%, which was not significantly different from the FEM model prediction (*P* > 0.05) (Fig. 2*F*). When different hypobaric pressures were applied to the skin an increase in pressure did produce a greater widening of the appendages, with a hypobaric pressure of 600 and 440 mbar generating a statistically significant increase in skin appendage diameter compared to the nonstretched skin (*P* < 0.05) (Fig. 2*F*). However, there was a plateau in the appendage opening effect, with 600 and 440 mbar not being statistically different from each other (*P* > 0.05). The depth of the skin appendages also appeared to decrease as the applied hypobaric pressure increased, and this agreed with the skin thinning shown in the modeling, but this trend was not as strong as the changes in appendage width, and it did not prove to be statistically significant across the series of measurements.

### Acyclovir, Tetracaine, and Diclofenac Diethylamine Formed Nanoaggregates through Self-Assembly in Aqueous Solutions.

To assess the ability of the patch to deliver large molecules directly into the skin, we decided to use drug nanoaggregates and labeled dextran macromolecules. Using drug nanoaggregates to test the ability of the patch to deliver large molecules into the skin afforded the opportunity to assess the administration of the monomeric drugs, presented to the skin using a concentration below their critical aggregation concentration, and nanoaggregates, using a concentration above their critical aggregation concentration (*SI Appendix*, Fig. S3). The skin permeation of both the drug monomers and nanoaggregates could be tracked using traditional permeation cells with liquid chromatography detection. Photon correlation spectroscopy was used to show that the drug monomers aggregated to form nanoaggregates at 0.62 ± 0.1 mM and 0.15 ± 0.02 mM for tetracaine and diclofenac diethylamine at pH 9 and 7.6 (these pHs were used to mimic the commercial topical product pH), respectively (*SI Appendix*, Fig. S3 *A–C*). No acyclovir self-association was detected at pH 7.6 (data not shown), but when the pH was changed to 5 (the pH of the skin surface) the acyclovir monomer aggregated to form nanoaggregates at 0.3 ± 0.02 mM (*SI Appendix*, Fig. S3*C*). Diclofenac diethylamine nanoaggregates were significantly smaller (*P* < 0.001) at 59.3 ± 10.2 nm compared to tetracaine at 190.2 ± 23.2 nm and acyclovir at 130.9 ± 17 nm (*SI Appendix*, Table S1). The relatively high polydispersity index (≥0.28) measured for all the test systems was indicative of the presence of different-sized nanoaggregates in the test solutions (*SI Appendix*, Table S1), but they each showed a monomodal size distribution in the light-scattering measurements. The zeta potential values obtained for diclofenac diethylamine (−9.19 ± 0.8 mV) indicated that a negatively charged nanoaggregate was formed, while the values were close to neutral for tetracaine (0.98 ± 0.5 mV) and acyclovir (−0.62 ± 0.3 mV) (*SI Appendix*, Table S1). The experimentally determined distribution coefficients for tetracaine (Log D 2.1 ± 0.16) and diclofenac diethylamine (Log D 0.58 ± 0.06) nanoaggregates were significantly lower (*P* < 0.05) when compared with the drug monomers (2.6 ± 0.12 and 0.8 ± 0.04, respectively) (*SI Appendix*, Table S1). This suggested that the self-assembly of the drugs into nanoaggregates enabled the agents to have more favorable interactions with the aqueous vehicle compared to the drug monomers, which accords with the traditional understanding of such agents self-assembling in water (24). The same trend was not seen for acyclovir suggesting the thermodynamic drive to aggregate was weaker.

### The Patch Enhanced the Skin Delivery of Diclofenac Nanoaggregates via the Appendages.

**Fig. 3.**
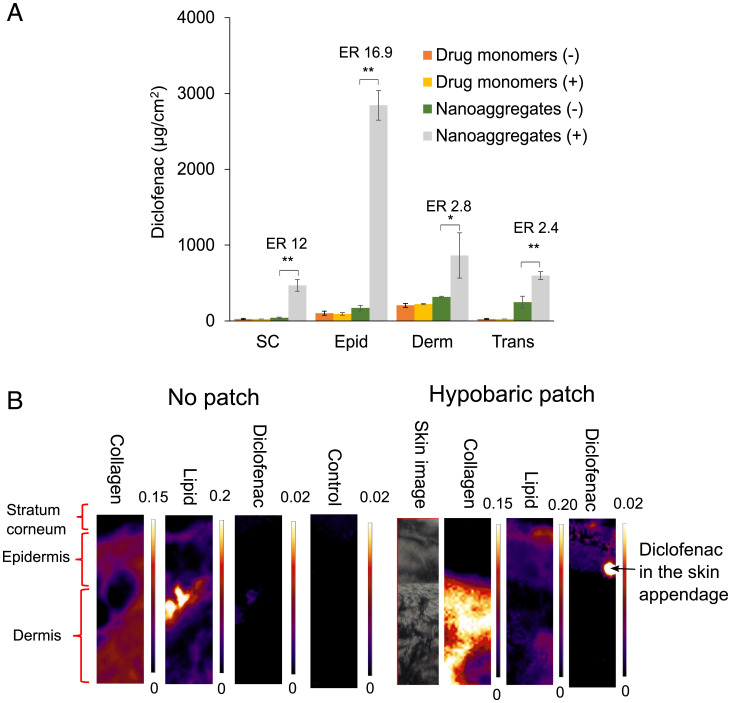
In vitro percutaneous penetration of (*A*) diclofenac diethylamine gel using porcine skin over 24 h with (+) or without (−) the application of the hypobaric chamber patch. Two concentrations were used: 0.5 mM when the drugs were monomers and 151 mM when the drugs were nanoaggregates. Data represent the mean ± SD (*n* = 5). ER represents the ratio between the amount of drug found using the patch vs. without the patch when the drugs were presented as nanoaggegates. Epid is the epiderms, Derm is the dermis, and Trans is transdermal. Student’s *t* test with *P < 0.01 and **P < 0.001. (*B*) The infrared mapping images of the skin with and without the application of the patch containing the hypobaric chamber showing collagen (based on 1,341-cm^−1^ peak, integration range 1,355 to 1,325 cm^−1^), lipid (based on 2,851-cm^−1^ peak, integration range 2,860 to 2,840 cm^−1^) and diclofenac (based on 1,306-cm^−1^ peak, integration range 1,315 to 1,295 cm^−1^) distribution in the skin lateral slices.

We next performed ex vivo deposition studies in porcine skin where acyclovir, tetracaine, and diclofenac diethylamine were applied topically as either drug monomers (0.5 mM) or nanoaggregates (151 mM) to determine the effects of the hypobaric chamber in the patch on skin delivery. The drug monomers showed no changes in skin deposition or in their transdermal permeation upon application of the hypobaric chamber (*P* < 0.05) (Fig. 3 and *SI Appendix*, Fig. S4). In contrast, concurrent nanoaggregate and hypobaric chamber patch application modified the delivery of all the drugs into the skin, but to differing extents (Fig. 3 and *SI Appendix*, Fig. S4). For acyclovir and tetracaine, the changes were significant, but modest, with enhancement ratios reaching 2.7-fold and 9-fold respectively (*SI Appendix*, Fig. S4). However, diclofenac was shown to have a 16.9-fold increase in the epidermal deposition and doubling of the transdermal penetration when the nanoaggregated drug was applied with the patch (Fig. 3). In addition, the patch modified the skin biodistribution of the three nanoaggregate therapeutic agents; for example, without the patch diclofenac was administered into the dermis and transdermally, but this changed to a greater epidermal targeting when the patch applied hypobaric pressure to the tissue (Fig. 3 and *SI Appendix*, Fig. S4).

As the confocal imaging data and FEM modeling suggested that the patch opened the skin appendages, Fourier transform infrared (FTIR) mapping of the skin slices was performed to search for diclofenac accumulation in the appendages during delivery. The skin was sectioned to view its three main layers, the SC, the epidermis, and the dermis. Within these layers, the infrared imaging results showed the characteristic infrared bands for water, lipid, and protein (amide I, amide II, and amide III) structures within the skin (25). As expected, the specific band of collagen at 1,341 cm^−1^, assigned to the CH_2_ side-chain rotation (proline) (26), was most abundant in the epidermis and dermis of the skin, whereas the lipid CH_2_ symmetric stretching mode at 2,851 cm^−1^ was most abundant in the SC (Fig. 3) (27). The differences in the skin collagen and lipid band intensities observed when the patch was applied were a consequence of the tissue stretching, but these changes did not prevent the tracking of diclofenac into the skin because it was not detected in the untreated skin or when only the diclofenac nanoaggregates were applied to the tissue. However, when the hypobaric chamber patch and the diclofenac nanoaggregates were applied simultaneously a strong diclofenac signal was observed in the SC and epidermis with a particularly strong spot in the region of the skin appendages, which indicated that the patch facilitated drug entry into the skin via the appendageal route (28).

### Macromolecules with a Molecular Weight of between 10,000 and 150,000 Da Show Enhanced Skin Delivery Using the Patch.

Next, fluorescein isothiocyanate (FITC)–labeled dextran, a model macromolecule, was used to understand the potential for the hypobaric patch to enhance the skin penetration of proteins and other biological agents. The size of dextran ranged from 11 to 24 nm in aqueous solutions and was not affected by the change in the pH of the solution or by an increase in temperature (*P* > 0.05) (*SI Appendix*, Table S2). Size-exclusion chromatography was employed to purify the material before the skin deposition studies (*SI Appendix*, Fig. S5). After one cycle of size-exclusion chromatography, to purify the 10-kDa and 70-kDa dextran the free FITC was removed and the molecular size distribution shifted to a slightly higher molecular weight (*SI Appendix*, Fig. S6). The 150-kDa FITC-dextran only required filtration ultracentrifugation to achieve the same result. These purification steps removed all the impurities according to the thin-layer chromotography analysis (*SI Appendix*, Fig. S7) and it resulted in a significant reduction (*P* < 0.001) in the FITC-dextran deposition and permeation across the skin. However, the general trend of reduced penetration of the dextran with increasing molecular weight, when applied to the tissue without the use of the hypobaric patch, was maintained (*SI Appendix*, Fig. S8 *A–C*).

**Fig. 4.**
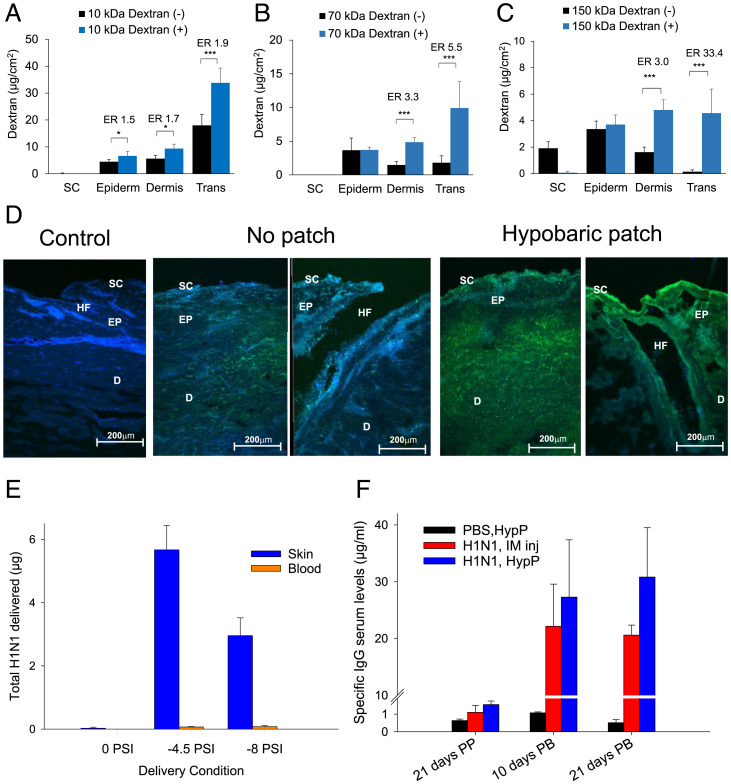
(*A–C*) The delivery of FTIC-dextran with (+) and without (−) the patch using a hypobaric pressure of 500 mbar: (*A*) 10 kDa FITC-dextran, (*B*) 70 kDa FITC-dextran, or (*C*) 150 kDa FITC-dextran. Skin deposition and permeation data represent mean ± SD (*n* = 5). ER was the ratio of dextran in the tissue with vs. without the hypobaric chamber patch. One-way ANOVA statistics applied (*A–C*) with *P < 0.05 and ***P < 0.001. (*D*) Confocal microscopy imaging (20× objective) of the skin (control) and the skin after the application of FITC-dextran (label is green) 10 kDa (*Left*) and 150 kDa (*Right*) with and without the hypobaric chamber patch. (*E*) The amount of H1N1 antigen delivered into the skin and blood noninvasively using the patch with two different application protocols data represents mean + 1 SD, *n* = 3 with *P < 0.05. (*F*) IgG response to the H1N1 antigen delivery into the skin using −4.5 psi for 20 min; data are mean + 1 SD, *n* = 3 with *P < 0.05.

The permeation experiments, which compared the effects of the patch on the dextran passage into and through the skin, demonstrated that the penetration into the dermal tissue and passage through the tissue transdermally were the most affected by the application of the patch to the skin as shown by the enhancement ratios (Fig. 4 *A–C*). The patch increased the skin penetration of the highest-molecular-weight dextrans more than the lower-molecular-weight molecules; e.g., the increase in transdermal skin penetration was 1.9-fold for 10 kDa, 5.5-fold increase for 70 kDa, and 33.4-fold increase for 150 kDa, when using the purified material (Fig. 4 *A–C*). Confocal images showed a greater dextran penetration into the skin when hypobaric chambers were applied to the tissue (Fig. 4*D*). The localization of the fluorescence in the images around the hair follicles suggested that application of the chamber encouraged dextran penetration through the appendages. However, strong signals from the dextrans were also recorded in the epidermal and dermal regions of the skin when the patches were applied, which suggested that the molecules were not trapped within the appendageal structures but rather passed out of the appendages and were available for subsequent pharmacological action (Fig. 4*D*).

### Needleless Hypobaric Patch-Administered H1N1 HA Antigen Provided a Superior Immunoglobulin G (IgG) Response Compared to Intramuscular (IM) Administration.

The FITC-labeled dextran experiments were used to select the delivery parameters for the administration of an H1N1 HA antigen using the hypobaric patch. Almost 6 µg of the antigen was administered into the mice with a hypobaric pressure of −4.5 psi for 20 min, but this was significantly reduced when a pressure of −8 psi for 5 min was employed (*P* < 0.05) (Fig. 4*E*). Very little of the antigen was delivered into the skin without the patch upon needleless delivery (Fig. 4*F*). The IgG response of the mice after two doses of the antigen compared to baseline showed that the delivery of the antigen via the patch was superior to the IM injection 21 d after the booster dose. The IgG concentrations were substantial at 20 µg/mL, exceeding those that have previously been reported with microneedle delivery (29).

### The Patch Delivered Diclofenac Nanoagregates to Effectively Reverse In Vivo Skin Inflammation.

**Fig. 5.**
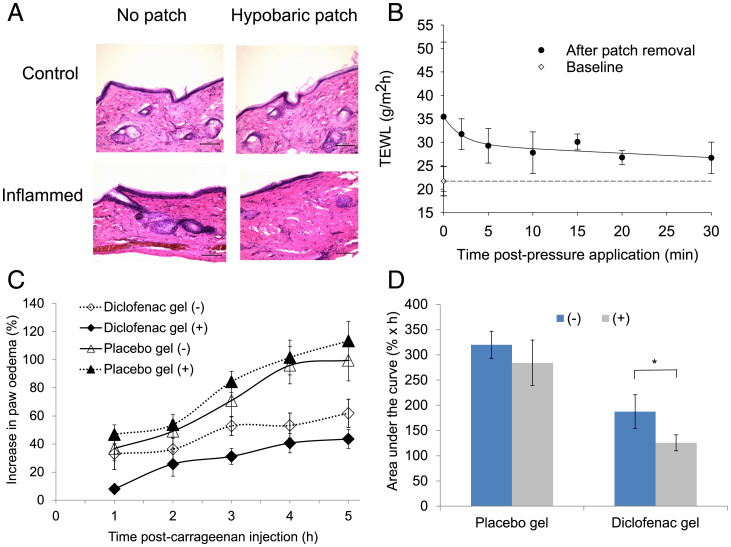
(*A*) Skin histology with and without the hypobaric patch application in healthy and inflamed rat paw skin. (*B*) TEWL after the application of the hypobaric patch. (*C*) Antiinflammatory activity of diclofenac diethylamine nanoaggregates formulated in a gel (43 mM) on rat carrageenan-induced paw edema without (−) and with (+) the patch. (*D*) The area under the curve of the swelling data *P < 0.001 (Mann–Whitney *U* test or analysis of variance, Bonferroni post hoc test); data are the mean ± SD (*n* = 5).

After demonstrating the needleless delivery of the H1N1 antigen achieved an effective immune response using the patch, the efficacy of the diclofenac nanoaggregates when administered directly to inflamed skin was tested. The skin appearance and histology before and immediately after patch application both in healthy and inflamed tissue demonstrated that 500 mbar of hypobaric pressure did not damage the tissue (Fig. 5*A* and *SI Appendix*, Fig. S9). The measurement of transepidermal water loss before the patch application and at various time points after application showed that the skin barrier disruption was temporary, with water loss returning to almost normal levels 30 min after the patch was removed (Fig. 5*B*). The intraplantar injection of carrageenan into the rat paws led to a time-dependent increase in paw edema (Fig. 5*C*). The edema was reduced upon the topical application of the diclofenac nanoaggregate gel. The coadministration of the patch significantly increased this antiinflammatory effect after 1 h and this enhanced effect was sustained over the 5-h study (*P* < 0.05; Fig. 5*C*). The application of diclofenac using a patch reduced the skin inflammation by 70% over the 5-h experiment and this was significantly more than the 50% reduction in edema when diclofenac was applied alone (Fig. 5*D*; *P* < 0.05).

## Discussion

This study applied a patch containing a chamber loaded with a pharmaceutical formulation to the surface of the skin. When the chamber was depressurized, the hypobaric conditions induced skin dome formation, and this opened the tissue appendages. The stretching of the skin reported in this work was in keeping with previous measurements in the literature with regard to both the skin dome heights and appendage diameters, which provided confidence that the tissue was naturally responding to the applied stresses (30, 31). However, the FEM modeling provided an important insight into the distribution of the stress in the tissue, which was not homogenous, and resulted in tissue compression in the upper regions of the interappendageal epithelium. To our knowledge, this has not previously been reported (30–34). It was an important finding as this skin compression provided a means to modify the interappendage epithelium and facilitate direct delivery into the viable tissue (12).

The ability of the patch to modify the penetration of small-molecular-weight drug molecules, drug nanoaggregates, and macromolecules into the skin was tested ex vivo. Interestingly, in the experiments employed in this work, the patch did not enhance the permeation of tetracaine, diclofenac, and acyclovir when presented to the skin in their monomeric form (their molecular weights ranged from 200 to 300 Da). If the hypobaric tissue stretching acted to permeabilize the skin in a manner similar to the application of temporal pressure, which reduces the skin barrier function, then the patch should have enhanced the penetration of these small molecules to some extent when not aggregated (12). However, the patch only enhanced the permeation of the drugs when forming nanosized aggregates that were more than 200 times larger than the individual molecules and would naturally be localized in and around skin appendages. Of the three drugs, the penetration of diclofenac was enhanced the most using the patch, and it displayed the best epidermal targeting. Diclofenac also formed the smallest and the most stable aggregates, which could have facilitated effective passage into the skin appendages once opened by the application of the hypobaric pressure patch (24, 35–38). The FTIR mapping studies confirmed that diclofenac accumulated in the skin appendages, and this accorded with previous work which has identified the skin appendages to be the primary skin penetration route for nanosized systems up to 600 nm (39, 40).

The size of the dextrans was about 10-fold smaller compared to the drug nanoaggregates when measured using dynamic light scattering of the unlabeled material [unlabeled dextran analysis was necessitated due to the FITC disturbing the laser (41)]. Initial attempts to assess the permeation of FITC-dextran into porcine skin suggested that considerable amounts of the macromolecules entered the skin without the patch application, but the subsequent removal of the free FITC label reduced this deposition (∼10 µg/cm^2^ for the 10-kDa FITC-dextran) to within the ranges previously reported in the literature (0∼2 µg/cm^2^ for a ∼4-kDa FITC-dextran) (42, 43). The skin penetration of purified FITC-dextran was enhanced by the application of the patch and the enhancement magnitude was much greater than that observed for the drug-rich nanoaggregates (up to 33-fold). The ability to load the skin with around 15 µg/cm^2^ of a 150,000 molecular weight model molecule suggested that the patch could be used to administer a protein-based vaccine in humans even when considering the different barrier properties of porcine vs. human skin. The confocal images suggested, like the FTIR mapping data, that the dextrans utilized the appendageal route to enter the skin, but in addition the confocal images indicated that the dextrans, once in the skin, passed out of the appendages and into the viable epidermis and dermis. This was confirmed by the needleless delivery of the H1N1 HA vaccine antigen into the skin and the resultant superior antigen-specific H1N1 HA IgG response compared to the dose-matched IM comparator. The specific IgG response after the second dose of the antigen was 30 µg/mL and this was approximately one order of magnitude higher than previous work with a soluble hemagglutinin antigen delivered by microneedles (29). This suggests that skin stretching could hold promise for self-vaccination, although the method of administration is in its infancy compared to microneedles, which have already shown promise in clinical testing (44).

The in vivo utility of the patch was further confirmed using a carrageenan-induced paw edema model with the developed diclofenac nanotherapeutic (45–47). The pharmacological action of topical diclofenac diethylamine nanoaggregates in the model was rapid and was believed to be dependent upon the tissue localization and action via the hypernociceptive cytokine cascade rather than a systemic effect (48–50). In this cascade, diclofenac acts to inhibit the prostaglandins, triggered by tumor necrosis factor α–mediated interleukin 6 and interleukin 1β release in the tissue. The diclofenac delivered using the patch was more effective in blocking this pathway compared to the same drug delivered by simple topical application (51). In the in vivo studies, the diclofenac nanoaggregates were not presented to the skin on the palm of the paw (glabrous paw skin) because there are no skin appendages in this region. Instead, they were presented to the nonglabrous skin on the back of the hind paw to allow the patch to act similarly to the ex vivo skin studies (17). This modification in the drug application method provided a stronger effect, reducing the inflammation by up to 70%, compared to previously published studies that employed a similar methodology (a 20% reduction in inflammation, using the front of the paw), probably because of the thinner skin and the availability of appendages on the back of the paw (52). Transepidermal water-loss measurements on the rat paw suggested that the patch increased the permeability for around 30 min after the application of the hypobaric pressure, then the normal barrier function returned. This reversal of the tissue-permeabilizing effects suggested that the patch can be used to repeatedly dose agents in the treatment of chronic diseases.

## Materials and Methods

### Materials.

Acetonitrile and methanol were high-performance liquid chromatography (HPLC) grade. Clear glass HPLC vials with crimpable lids and 0.45-µm nylon filter papers, SlowFade Gold Antifade Mountant with DAPI, and optimum cutting temperature (O.C.T.) solution were all purchased from Fischer Scientific. Tetracaine base and aciclovir base were both BP grade (99.9%). Isofluorane, isopentane, carrageenan, sterile saline, hematoxylin (Gill No3) solution, eosin solution, and differentiating solution were supplied by Sigma-Aldrich. Diclofenac diethylamine BP grade (99.9%) was from Unique Chemicals. Certified dextran standards (12 kDa, 80 kDa, and 150 kDa) were supplied by Merck Life Science. Concentrated hydrochloric acid and sodium hydroxide were from Fluka. Sodium acetate was provided by Alfa Aesar. Deionized water was obtained by purification using an Elgstat water purifier (Elga Ltd). Hydroxypropyl methylcellulose grade 65SH viscosity 50 cP with the brand name Metolose was supplied by Shin-Etsu Chemical Ltd.

#### FEM Simulations.

COMSOL Multiphysics v5.5 was used to obtain the numerical FEM results in this work. A three-dimensional geometry (a rectangular domain with dimensions 32 mm × 32 mm × 1.53 mm) was created to reproduce the hypobaric skin experiments (Fig. 2*C*). The Solid Mechanics module was used. A one-term Ogden hyperelastic model with the shear modulus (μ=0.03 MPa and alpha parameter α=13.57) was used to describe the mechanical properties of the material. A complete description of the FEM model used to describe the skin biomechanics can be consulted in ref. 53. The boundary conditions are as described in Fig. 2*A*. The hypobaric pressure is applied over the central circular area, which has the same inner diameter as the glass vacuum chamber (8 mm). The thickness of the glass chamber is not considered. The model assumes no initial compression on the skin membrane caused by the positioning of the glass chamber (53). The lateral boundaries are fixed to mimic the effect of the pins that attach the skin to the board. The rest of the upper boundary (excluding the central circular area) is also fixed to mimic how the glass chamber blocks its movement. The central circular area and the lower boundary can experience free lateral and vertical movement. The results were all obtained by using a Physics Controlled mesh with the option Extra Fine, which gave, as a result, a mesh comprised of 14,410 tetrahedra. The relative tolerance of the solver was set at 10^−8^. The simulations were run in static mode.

#### Photon Correlation Spectroscopy Characterization.

Changes in derived count rate were tracked using PCS (Malvern Nanoseries Zetasizer; Malvern Instruments Ltd). Measurements were taken at a scattering angle of 173°. The refractive index and viscosity constants were set at 1.33 and 0.88 mPa⋅s, respectively. Samples were filtered through a 0.45-µm cellulose nitrate filter before the analysis. The scattering information was determined in an aqueous vehicle (acetate buffer 0.1 M) at increasing molar concentrations using the pH of the commercial topical products: pH 9 for tetracaine and pH 7.4 for diclofenac diethylamine and aciclovir. Additional studies at pH 5 were performed for acyclovir as preliminary experiments had suggested that the molecule aggregated more effectively at this pH. Control solutions were prepared in the same manner as for the test systems but without the addition of the drug. A discontinuity in the linear models applied to the data indicated the formation of a nanosized aggregate structure in the solution. The concentration at which this change occurred, confirmed by the application of a second derivative function (OriginPro 9.1 Software; OriginLab), was defined as the critical aggregation concentration. The size of the molecular aggregates was determined by converting the light scattering signal into a hydrodynamic radius using the Stokes–Einstein equation (Eq. **1**) where *D* was diffusion coefficient, *K_B_* Boltzmann’s constant, *T* was the temperature (Kelvin), *η* was solvent viscosity, and *R*_0_ was particle radius. The zeta potential of the aggregate containing solutions was recorded in the same vehicle employed for the molecular aggregate analysis and the concentration of each drug was set above critical aggregation concentration at pH 7.4 for diclofenac diethylamine, pH 5 for aciclovir, and at pH 9 for tetracaine.

[1] $$D=\frac{K_{B}T}{6\pi R_{0}\eta }$$

The size of the dextrans (12 kDa and 150 kDa) was obtained using the Zetasizer at 24 °C (formulation temperature) and 32 °C (skin temperature) using phosphate-buffered saline (PBS) at pH 7.4 and pH 5 to mimic the formulation and the skin conditions, respectively. The concentration for each tested dextran was 0.5% wt/vol (which was selected based on the findings from the method optimization work). Methods were previously described in ref. 54.

#### Apparent Distribution Coefficient.

The apparent drug distribution coefficients (Log D) were measured at room temperature, below and above the critical aggregation concentration in a two-phase *n*-octanol and acetate buffer (0.1 M) system at pH 7.4 for diclofenac diethylamine, pH 5 for aciclovir, and pH 9 for tetracaine, following a previously described method (55). After phase separation, the aqueous phase was withdrawn and samples were centrifuged at 13,000 rpm (Biofuge; Heraeus). Aliquots of the liquid phase were transferred into vials and analyzed by HPLC and the apparent distribution coefficient was calculated using Eq. **2** where *c*_0_ was the concentration in the organic layer, *c_i_* was the concentration of ionized species in water, and *c_u_* is the concentration of unionized species in water. Methods were previously described in ref. 54.

[2] $$D=\frac{C_{0}}{\;(C_{i}+C_{u})w}$$

### Formulation Preparation.

Hydroxypropyl methylcellulose (HPMC) was selected as the gelling agent because it is a nontoxic, nonionic, inert polymer which has been extensively used in the preparation of topical pharmaceutical formulations. A 3% HPMC gel was prepared with a drug load above and below the experimentally determined critical aggregation concentration for each test active. The concentration above the critical aggregation concentration was selected to match the concentration of the commercial product, and where possible, close to its saturated solubility in the vehicle. Gel formulations at concentrations of 0.5 and 151 mM and 0.12 and 43 mM were prepared for tetracaine and diclofenac diethylamine, respectively. Conversely, since no significant aggregation was detected at the pH of the acyclovir commercial product (pH 7.4, 222 mM), 0.15 and 2 mM formulations were prepared at pH 5 because this pH showed the presence of aggregates. The gel was prepared by heating 25 mL of distilled water up to 70 °C, adding 1.5 g of Metolose, and stirring until complete dissolution of the polymer had occurred. The drug was prepared in a second aqueous solution, and both phases were combined and then stirred homogeneously on a stirring plate (Stuart Scientific). The mixture was cooled in a refrigerator to *ca*. 4 °C until it became transparent. The pH was then adjusted to the required value by adding NaOH (1 M) or acetic acid as required and then the mixture was stirred for at least 30 min until room temperature was reached. The pH of the gel was then checked by diluting and dispersing it in water (10% wt/vol) in a manner described elsewhere (56). Visual inspection indicated that the formulated preparations remained physically stable for the entire duration of the experimental period and were found to be transparent and of uniform consistency. Methods were previously described in ref. 54.

#### Dextran Assay.

Quantitative determination of FITC-dextran in the skin permeation and deposition studies and for the purification studies was performed using a Spark plate reader (Tecan Ltd) at an excitation wavelength of 485 ± 20 nm and emission wavelength of 535 ± 20 nm. The diluent used was identical to that used for the permeation and purification studies (i.e., PBS, pH 7.4) and the volume of the solution per well was 200 µL. The calibration curves were constructed according to fluorescence intensity measurements. Calibration curves were obtained for the commercial FITC-dextrans as well as the FITC sodium salt. The assay for each of the tested (commercial and purified) FITC-dextrans was verified to be “fit for purpose” by determination of linearity, precision, and sensitivity. The effect of hypobaric pressure application on the dextran transdermal and skin deposition was expressed as enhancement ratio (ER) as per Eq. **3**:

[3] $$ER=\frac{C_{\mathrm{hypobaric}\;\mathrm{patch}}}{C_{\mathrm{without}\;\mathrm{the}\;\mathrm{patch}}},$$

where *C_hypobaricpatch_* was the amount of dextran deposition in the given skin layer (micrograms per square centimeter) under hypobaric pressure conditions and the *C_without the patch_* was the amount of dextran deposition in the given skin layer (micrograms per square centimeter) under atmospheric pressure condition.

#### FITC-Dextran Purification before Permeation Studies.

FITC-dextran (150 kDa) was purified using 12 consecutive cycles of ultrafiltration at 8,000 rpm for 10 min (Amicon filters, 100-kDa molecular weight cutoff [MWCO]). The obtained final solution was then analyzed using gel filtration size-exclusion chromatography (Sephadex G-100 19-cm column as the stationary phase and water as the mobile phase) to confirm the absence of any free FITC label. FITC-dextran, 10 kDa and 70 kDa, were purified using a gel filtration size-exclusion chromatography column and the obtained solutions (volume fractions 9 to 10 for the 10-kDa and 7 to 8 for the 70-kDa FITC-dextran) were concentrated using ultrafiltration (Amicon filters, 3 kDa MWCO, one cycle). The obtained concentrated FITC-dextran solutions were then reanalyzed using size-exclusion chromatography to confirm the absence of a free FITC label.

The commercial and purified FITC-dextrans were also analyzed with thin-layer chromatography using silica gel 60 RP-18 F_254_S plate (Merck Life Science). The plates were first developed with 80% methanol and 20% distilled water and dried; the original unpurified FITC-dextrans were fully resolved from their impurities using 20% methanol and 80% PBS pH 7.4. Developed plates were illuminated under a 366-nm ultraviolet lamp (Krackeler Scientific).

### Ex Vivo Skin Studies.

Full-thickness porcine skin was used in the ex vivo experiments as the appendages have been shown to remain open during permeation studies rather than close like in human skin (10, 15). The methods have been previously described in ref. 19 and are detailed in *SI Appendix*. The studies were initiated by the application of an infinite dose of each in-house formulated gel (1 g) or by the application of either the purified or commercial FITC-dextran solution (1 mL, 500 mg/mL in PBS, pH 7.4) to the apical surface of the porcine skin. The studies were conducted for 24 h under atmospheric pressure (1,010 mbar) or hypobaric pressure (500 mbar) applied for the first hour. The samples were maintained at room temperature until a quantitative determination by HPLC analysis (for diclofenac diethylamine, tetracaine, and acyclovir) and by fluorescent spectroscopy (for the 10-kDa, 70-kDa, and 150-kDa FITC-dextran). At the end of the in vitro skin permeation studies, the Franz diffusion cells were dismantled, the skin was removed and wiped with water, two tape strips were removed, and the test molecules extracted and quantified. The effect of hypobaric pressure-controlled skin stretching upon drug cutaneous tissue deposition was represented by an enhancement ratio (ER) which was calculated as per the dextran studies. Methods were previously described in ref. 54.

### HPLC Assay.

A liquid chromatography pump (P680 HPLC pump; Dionex) with an ASI-100 automated sample injector (Dionex) connected to a PDA-100 photodiode array detector (Dionex) was used for the quantitative determination of each drug molecule. The HPLC system was connected to a computer with Chromeleon software (Dionex), which was used to record and analyze the chromatograms. A Luna C18 (5 µm, 250 × 4.6 mm) column (Phenomenex) was used with a 50:25:25 and 35:25:40 acetate buffer (0.1 M)/methanol/acetonitrile mobile phase at pH 4 for tetracaine and diclofenac diethylamine and a 96:4 acetate buffer (0.1 M)/methanol mobile phase at pH 4 for aciclovir at a flow rate of 1 mL⋅min^−1^. Volumes of 50 µL were injected onto the column and tetracaine, diclofenac diethylamine, and aciclovir were analyzed at a wavelength of 311, 220, and 254 nm, respectively. The method was shown to be fit for purpose in terms of linearity (*R*^2^ > 0.999), peak symmetry (0.8 to 1.3), and sensitivity (the LOD for tetracaine, diclofenac diethylamine, and aciclovir was 3.26 µg⋅mL^−1^, 3.14 µg⋅mL^−1^, and 2.78 µg⋅mL^−1^ and the LOQ values were 9.76 µg⋅mL^−1^, 8.92 µg⋅mL^−1^, and 10.58 µg⋅mL^−1^, respectively) according to the limits described by the International Conference on Harmonization guidelines.

#### Confocal Microscopy.

Porcine skin was prepared and mounted in Franz cells as in the skin permeation studies. Permeation studies using 150-kDa FITC-dextran solution (500 mg/mL in PBS, pH 7.4) were conducted with and without the chamber patch (*n* = 3 for each) as was described earlier for the permeation studies. At the end of the permeation experiment, the skin was removed from the Franz cells and any remaining formulation on the surface of the skin was carefully removed by washing the skin five times using deionized (DI) water and cotton buds and by the application of two tape strips. The skin was cut into strips (three from each Franz cell), embedded in O.C.T., and frozen gently using chilled isopentane (isopentane was chilled to near freezing by immersing the isopentane container in liquid nitrogen) then cut into lateral tissue sections with a thickness of 20 µm using a cryostat (Bright Instruments). The tissue sections were mounted onto microscopy slides (SuperFrost; Fisher Scientific), washed in PBS (1 min), and fixed in 10% formalin (10 s) then mounted using SlowFade Gold Antifade Mountant, with DAPI, and sealed with a coverslip. Confocal microscopic images were obtained with A1 inverted confocal with spectral detector by Nikon at a magnification of 20×. Images were obtained with two fluorescence channels: DAPI (405 nm) and FITC (488 nm). Images were analyzed using NIS-Elements Imaging Software (Nikon).

### FTIR Mapping.

Porcine skin was prepared and mounted in Franz cells as in the skin permeation studies. A 3% HPMC gel was prepared with diclofenac loaded above its critical aggregation concentration, i.e., 43 mM, and applied to the skin for 24 h with and without the hypobaric chamber patch (500 mbar). At the end of the permeation experiment, the skin was removed from the Franz cells and any remaining formulation on the surface of the skin was carefully removed by washing the skin five times using DI water and cotton buds and by the application of two tape strips. The skin was cut into strips, embedded in O.C.T., and frozen gently using chilled isopentane (isopentane was chilled to near freezing by immersing the isopentane container in liquid nitrogen) then cut into five to nine lateral tissue sections with a thickness of 10 µm followed by mounting on a CaF_2_ substrate. The FTIR images were generated using a linear array FTIR microscope (Spotlight 400; PerkinElmer). FTIR images were collected in transmission mode with the following settings: 6.25-µm pixel size, 8-cm^−1^ spectral resolution, and an average of 32 scans. The FTIR mapping of the skin sections was performed by integrating the area under the band within the same spectral range specified with a straight baseline drawn between the integral limits. Bands of collagen (based on 1,341-cm^−1^ peak, integration range 1,355 to 1,325 cm^−1^), lipid (based on 2,851-cm^−1^ peak, integration range 2,860 to 2,840 cm^−1^), and diclofenac (based on 1,306-cm^−1^ peak, integration range 1,315 to 1,295 cm-1) were used to generate the images.

### Confocal Live Three-Dimensional Imaging.

A StageFlexer (Flexcell International) was used to view the real-time skin stretching under hypobaric pressure (765, 510, 310, and −24 mbar). The StageFlexer was prepared by inserting the loading station with freshly prepared porcine skin, stained with DAPI, on top of one another and securing the assembly using the retaining screws. The cell was mounted with a glass coverslip. The StageFlexer was connected to a handheld vacuum pump with a gauge (Fischer Scientific), which allowed the application of a hypobaric pressure in an identical manner to the ex vivo skin permeation studies. Confocal microscopic images were obtained with A1 inverted confocal with spectral detector by Nikon at a magnification of 10×. Images were obtained with two fluorescence channels: DAPI (405 nm) and FITC (488 nm). Images were analyzed using NIS-Elements Imaging Software (Nikon).

### In Vivo Delivery of Vaccine Antigen.

All procedures were conducted per the UK Animal Scientific Procedures Act (1986) and Amendments Regulations (2012) and approved by the King’s College London Animal Care and Ethics Committee. Female balb/c mice (7 to 9 wk old, *ca*. 19 to 22 g; Envigo) were caged in groups of four with free access to water and food. A temperature of 19 to 22 °C was maintained, with a relative humidity of 45 to 65% and a 12-h light/dark cycle. Animals were acclimatized for 7 d before each experiment.

The level of H1N1 delivered across the skin using the needle-free hypobaric patch was determined in vivo in balb/c mice under two pressure conditions: at −4.5 psi for 20 min or at −8 psi for 5 min. Briefly, the application the device chamber was applied on the shaved lower dorsal skin of the mice, the desired pressure level was induced using an automated vacuum pump system, and then the antigen formulation was inserted through the delivery port. At the end of the hypobaric pressure application time (either 20 min or 5 min), the chamber was removed and the formulation (1 mg/mL H1N1 antigen in sterile PBS, pH 7.4) was maintained on the application site with the aid of an application O-ring for a total application time of 1 h. Twenty-four hours later, blood and skin tissue samples were obtained. The serum was obtained and stored at −80 °C until analysis. The skin tissue was incubated overnight at 4 °C in a lysis buffer (Tris 80 mM, pH 8, NaCl 137 mM, Nonidet P-40 1% vol/vol, and 2mM ethylenediaminetetraacetic acid) and then homogenized at 4 °C. The homogenate was centrifuged and filtered (0.2 µm) before analysis of H1N1 HA content using a specific H1N1 HA enzyme-linked immunosorbent assay (ELISA) (assay sensitivity = 83 ng/mL, *R*^2^ = 0.997, tissue and serum interference with the assay was accounted for by subtracting the relevant background (serum or tissue lysate) from the sample absorbance.

### In Vivo Vaccine Efficacy Study.

Mice were randomly assigned to receive the H1N1 HA antigen formulation either through the skin using the needle-free hypobaric patch or IM injection into the leg muscle. The IM injection H1N1 HA dose matched the dose delivered into the skin as determined from the in vivo delivery work (i.e., ∼6 µg per mouse). In addition, there was a group that received a placebo (PBS buffer, pH 7.4) by the needle-free hypobaric pressure device. The hypobaric patch used pressure in the range of −4 to −5 psi for 20 min, then the pressure was removed. The antigen formulation remained on the skin for a further 40 min. All mice received two doses of the antigen or the control formulation at 3-wk intervals (dosing method adapted from ref. 57). Blood samples were obtained at baseline levels, before the second dose, and at 10 d and 21 d after the second dose. Serum was obtained and stored at −80 °C until analysis. Levels of specific IgG levels were determined in the serum samples using a fit-for-purpose ELISA that employed plated coating using the H1N1 HA antigen (limit of sensitivity = 1.1 ng/mL, *R*^2^ = 0.999).

### In Vivo Antiinflammatory Assay.

All procedures were conducted per the UK Animal Scientific Procedures Act (1986) and Amendments Regulations (2012) and approved by the King’s College London Animal Care and Ethics Committee. Sprague-Dawley male rats (6 to 9 wk old, *ca*. 220 to 250 g; Charles River) were caged in groups of four with free access to water and food. A temperature of 19 to 22 °C was maintained, with a relative humidity of 45 to 65% and a 12-h light/dark cycle. Animals were acclimatized for 7 d before each experiment.

The antiinflammatory activity of diclofenac diethylamine formulated in a hydroxypropyl methylcellulose gel with a drug load above critical aggregation concentration, as per the ex vivo permeation experiments, was studied in a rat carrageenan-induced paw edema model under atmospheric (1,010 mBar) and hypobaric (500 to 650 mBar) pressure conditions, i.e., without and with the hypobaric patch. Rats were randomly divided into six groups (*n* = 5) and the experimenter was blinded toward the different conditions applied to the ipsilateral hind paw at the time of the study. The contralateral paw was untreated for the duration of the experiments using the chamber which was either left at atmospheric conditions or hypobaric conditions. Animals were anesthetized by inhalation of (1 to 3%) isoflurane/(2%) O_2_ and placed on a heating mat (Harvard Apparatus) in the dorsal position maintained at 36 °C for the duration of the anesthesia. The contralateral and ipsilateral hind paw thickness in the dorsal plantar axis was measured with a caliper (Mitutoyo) and the point of measurement was premarked as a reference for subsequent measurements (58). The first and the second groups served as controls by the 2-µL topical application (equivalent to ∼5 µg/cm^2^) of an in-house–formulated placebo gel with and without the patch (depressurized at 500 mBar for 20 min). In the third and fourth groups, the formulated gel contained diclofenac diethylamine (43 mM), which was topically applied to the hind paw nonglabrous skin with and without the patch. Carrageenan paw edema was induced by subplantar injection (0.1 mL of 1% wt/vol carrageenan in 0.9% saline) after 20 min of topical application of the formulated gel and then the anesthesia was interrupted. Animals were recovered and changes in paw thickness were determined by triplicate measurements carried out at 1-h intervals up to 5 h after the carrageenan injection. All animals were culled by a schedule 1 method upon the termination of the experiment. The results were expressed as paw swelling % (PS) which was calculated according to Eq. **4**, where *T_e_* was the mean paw edema thickness at a specific time point after carrageenan-induced inflammatory response and *T_i_* was the initial paw thickness (basal value). Methods were previously described in ref. 58.

[4] $$PS=\frac{T_{e}-T_{i}}{T_{i}}\;\times \;100$$

### Histology Studies.

Skin biopsies were obtained from the untreated healthy paw and untreated inflamed paw at 6 h after intraplantar injection of the 1% carrageenan and the paw of animals challenged under the same hypobaric stress conditions employed in the antiinflammatory assay studies. The paw nonglabrous skin samples were fixed with 4% neutral-buffered formalin for 24 h at 4 °C then were cut in strips, embedded in O.C.T., and frozen as described for the confocal imaging section. Cross-section slices of 10-µm thickness were obtained using a Bright Model OTF cryostat (Bright Instruments). The samples were then stained following the Ellis hematoxylin and eosin staining protocol (57) and dehydrated with different volumes of ethanol (75%, 95%, and 100%) and xylene before being mounted in DPX and covered with glass coverslips. The samples were analyzed using a Leica DM 200 LED light microscope (Leica Microsystems) equipped with a Leica digital camera (model DFC 295) at a magnification of 20×. Images were processed using Las v4.4 Imaging Software (Leica Microsystems).

### Transepidermal Water-Loss Measurement.

The transepidermal water loss (TEWL) across the paw’s skin was measured in vivo in Sprague-Dawley rats with and without the hypobaric patch using a method identical to that used for the antiinflammatory assay. TEWL was obtained on untreated paws (baseline measurement) and those treated with the patch for 20 min using an AquaFlux Model AF200 TEWL meter (Biox Systems Ltd).

### Statistical Data Analysis.

Statistical evaluation was carried out using a statistical package for social sciences software (SPSs version 16.0; SPSS Inc.). All data were checked in terms of normality (Kolmogorov–Smirnov test) and homogeneity of variances (Levene’s test) before analysis. Statistical analysis was performed using Student’s *t* test, Mann–Whitney *U* test, or two-way analysis of variance followed by Bonferroni’s comparison post hoc test. In all cases, a statistically significant difference was defined as when *P* < 0.05 and denoted as *P < 0.05, **P < 0.01, and ***P < 0.001. The number of replicates was five in the ex vivo permeation studies and antiinflammatory assay and three in the apparent distribution coefficient determination and light-scattering studies.

## Supplementary Material

Supplementary File

## Data Availability

All study data are included in the article and/or *SI Appendix*.
