# Supplementary material for: Needleless administration of advanced therapies into the skin via the appendages using a hypobaric patch
Source: Proc Natl Acad Sci U S A. 2022 Apr 28;119(18):e2120340119. doi: 10.1073/pnas.2120340119 (PMC9170139; doi:10.1073/pnas.2120340119)
Supplement: Supplementary File [file pnas.2120340119.sapp.pdf]

PNAS
www.pnas.org

Supporting Information

Needleless administration of advanced therapies into the skin via the appendages using a hypobaric patch

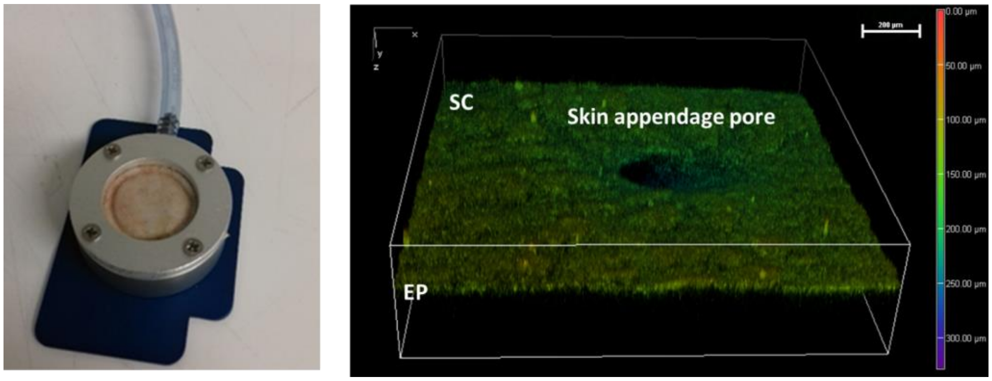

Figure S1. Left. The StageFlexer® unit that was used in the confocal imaging of the skin hair follicles mounted with full thickness skin. Right. A 3-D view of full thickness porcine skin mounted on the StageFlexer showing a single skin appendage pore and acquired using confocal microscopy (x10). Colour spectrum indicates the depth of the tissue layer with the stratum corneum (SC) facing upward and the viable epidermis (EP) towards the bottom of the image. Tissue dimensions; W 1272.79 mm, H 1272.79 mm, D 328.22 mm.

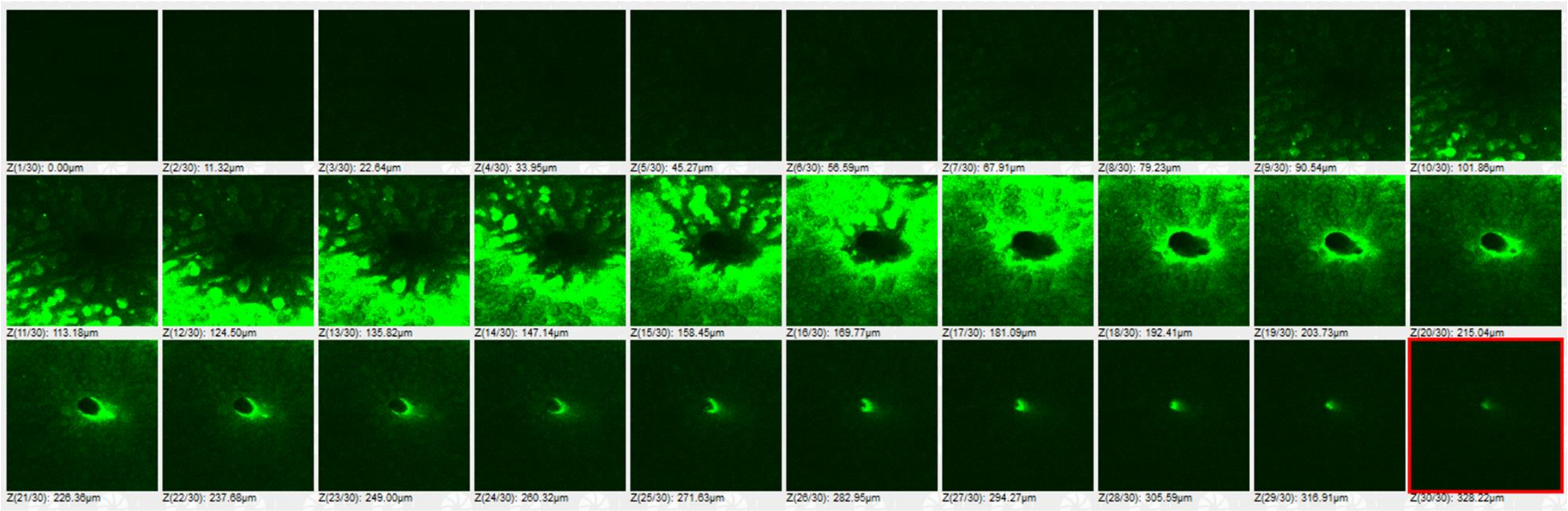

Figure S2. Confocal images of full thickness porcine skin mounted on the StageFlexer and showing a skin appendage pore at different z-levels (x10). Green colour arises from the autofluorescence of the skin tissue.

a)

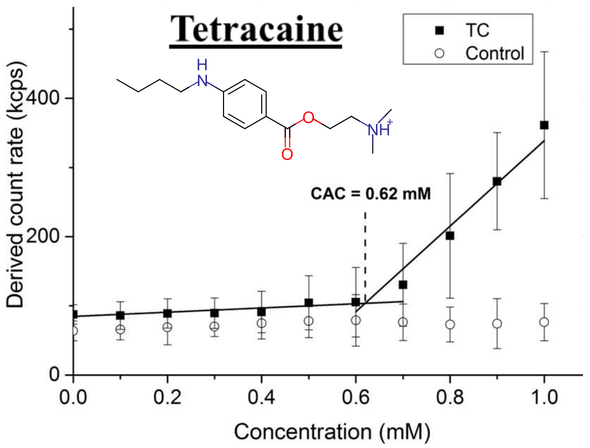

b)

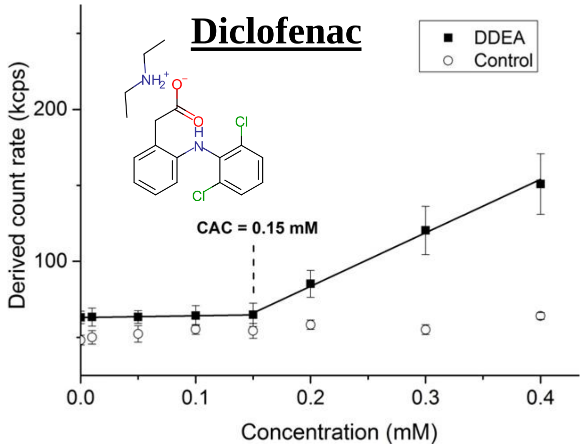

c)

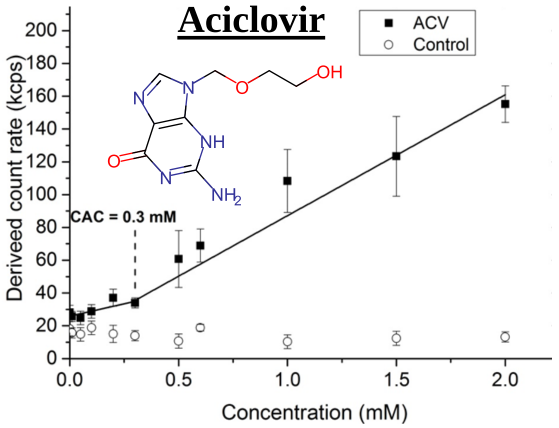

Figure S3. The aggregation of three drugs in buffer, a) tetracaine (TC) at pH 9 and b) diclofenac diethylamine (DDEA) at pH 7.6 c) aciclovir (ACV) at pH 5. CAC refers to critical aggregation constant.

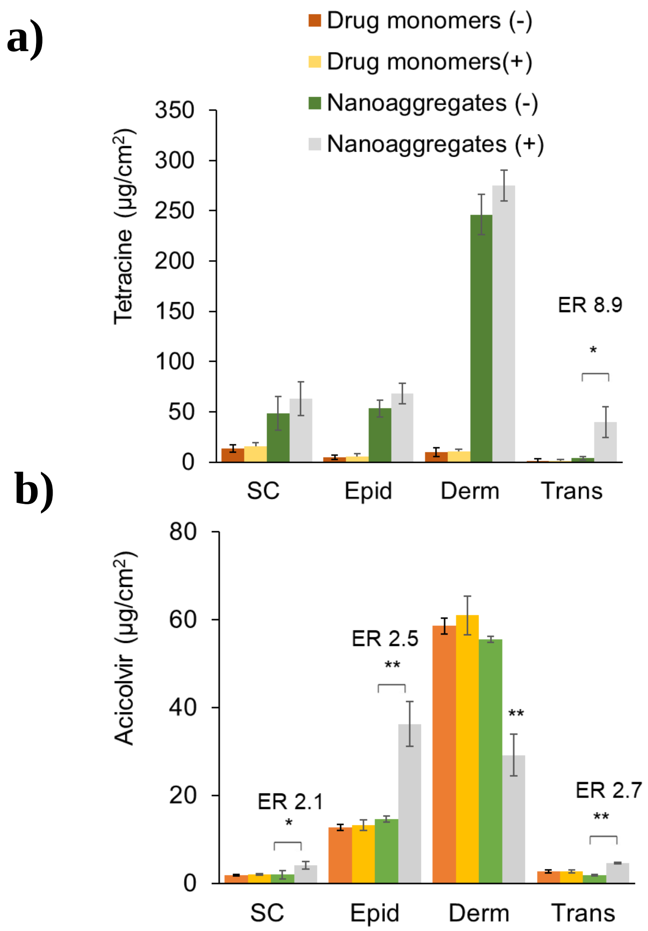

Figure S4. *In vitro* percutaneous penetration of (a) tetracaine gel and (b) acyclovir gel using porcine skin over 24 h with (+) or without (-) the application of the hypobaric chamber patch. Two concentrations were used 0.5 mM when the drugs were monomers and 151 mM when the drugs were nanoaggregates. Data represents the mean ± standard deviation (n = 5). ER (Enhancement ratio) represents the ratio between the amount of drug found using the patch vs without the patch when the drugs were presented as nanoaggegates. SC is stratum corneum, Epid is the epiderms, Derm is the dermis and Trans is transdermal. Student's t-test with * $p < 0.01$ and ** $p < 0.001$.

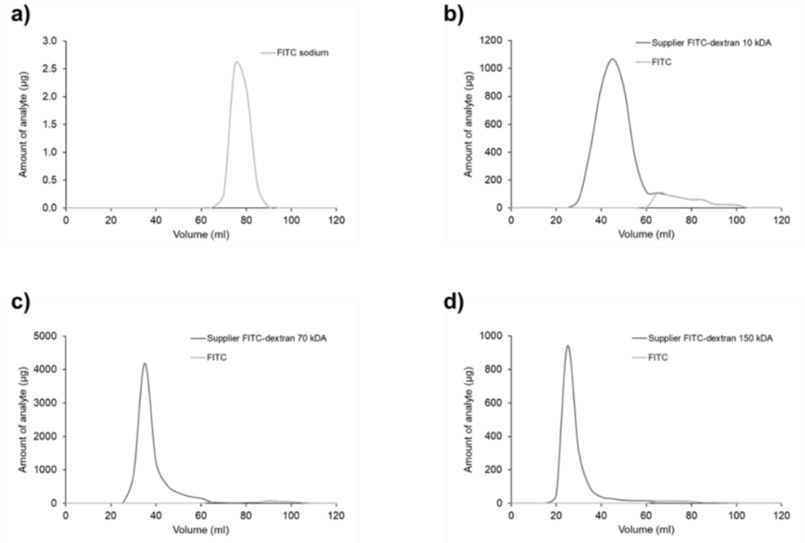

Figure S5. Size exclusion chromatography of a) FITC sodium, supplier FITC-dextran b) 10 kDa, c) 70 kDa and d) 150 kDa. The amount of free-FITC label was calculated based on peak of FITC sodium which starting eluting at volume 65 ml.

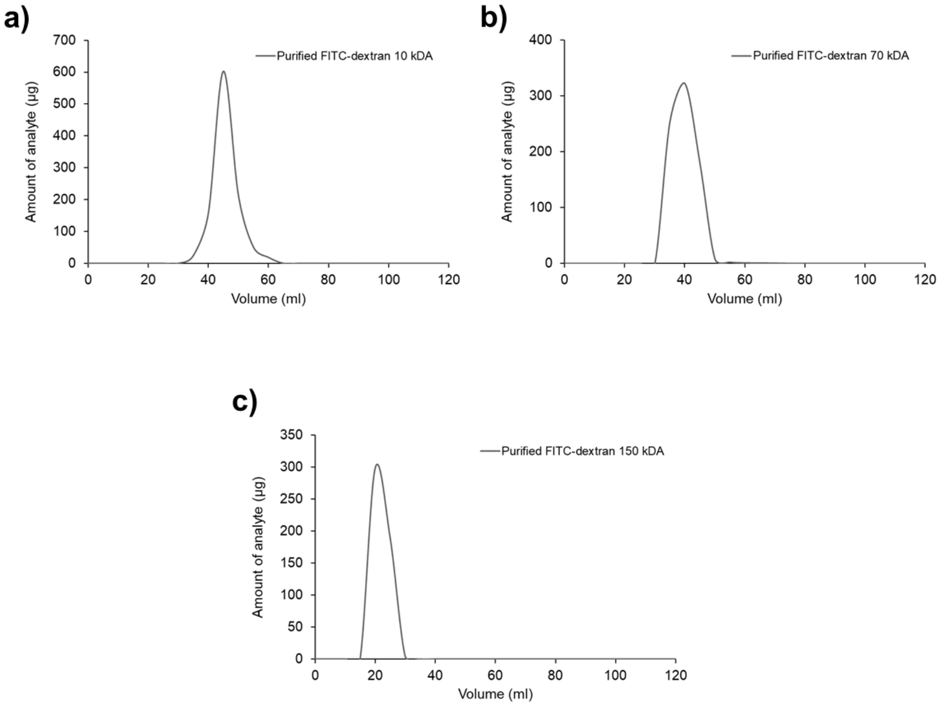

Figure S6. Size exclusion chromatography of purified FITC-dextran a) 10 kDa, b) 70 kDa and c) 150 kDa.

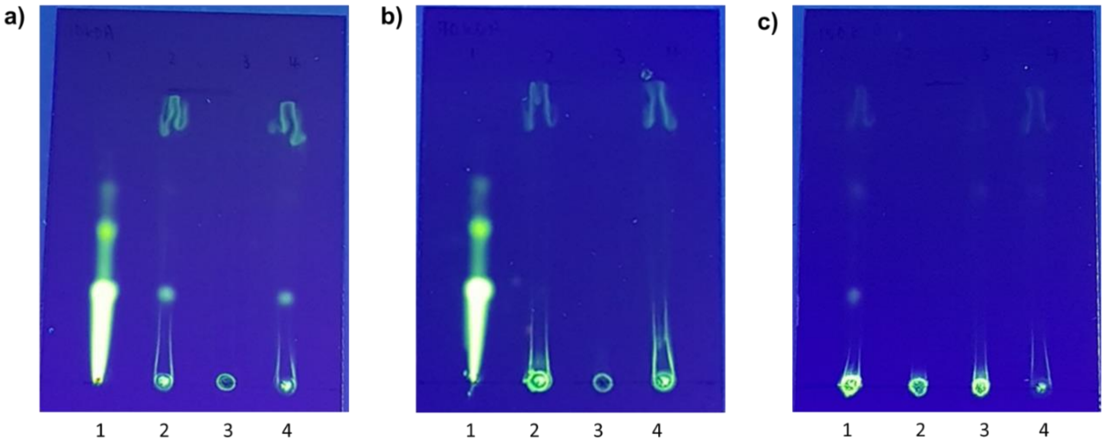

Figure S7. Thin layer chromatography of FITC-dextran with varying degrees of purity. a) lane 1 – FITC 500 µg/ml, lane 2 – supplier FITC-dextran 10 kDA 2 mg/ml, lane 3 – purified FITC-dextran 10 kDA 500 µg/ml (with Sephadex G100 column and ultra-filtration), lane 4 – FITC-dextran 10 kDA 2 mg/ml with one cycle of ultra-filtration; b) lane 1 – FITC 500 µg/ml, lane 2 – supplier FITC-dextran 70 kDA 2 mg/ml, lane 3 – purified FITC-dextran 70 kDA 500 µg/ml (with Sephadex G100 column and ultra-filtration), lane 4 – FITC-dextran 70 kDA 2 mg/ml with one cycle of ultra-filtration; c) lane 1 – supplier FITC-dextran 150 kDA 2 mg/ml, lane 2 – purified FITC-dextran 150 kDA 500 µg/ml (with 12 cycles of ultra-filtration), lane 3 – FITC-dextran 2 mg/ml with one cycle of ultra-filtration, lane 4 – filtrate of FITC-dextran 150 kDA ultra-filtration.

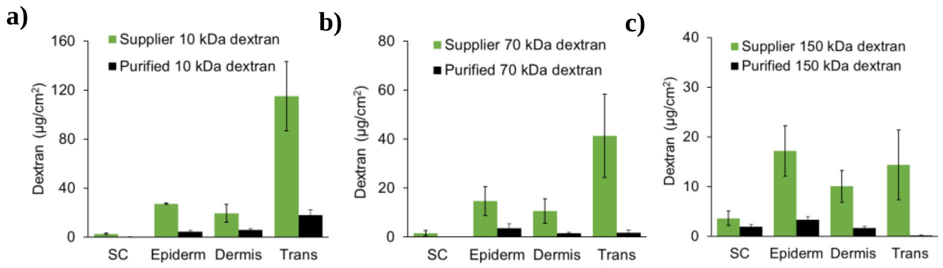

Figure S8. The effect of FITC-dextran purification on the skin deposition and of a)10 kDa b) 70 kDa and c) 150 kDa at atmospheric pressure over 24 h.

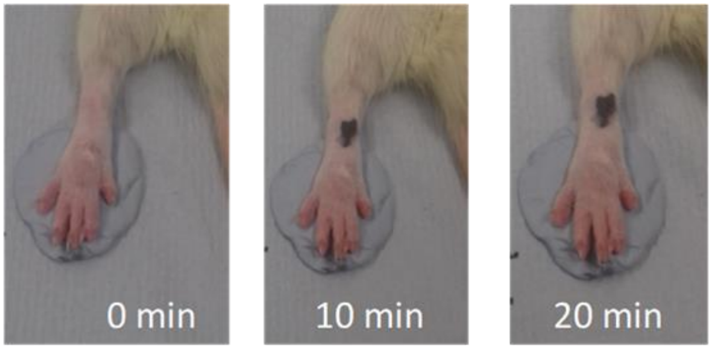

Figure S9. Image of the rat paw immediately, 10 min and 20 min after the application of the hypobaric patch.

Table S1. The characteristics of the drug aggregates. [a] indicates the hydrodynamic size, [b] polydispersity index, [c] post aggregation apparent distribution coefficient and *pre-aggregation apparent distribution coefficient. Each point represents the mean ± standard deviation (n = 3).

**Drug**	**pH**	**Size [a] (nm)**	**P.I. [b]**	**Zeta potential (mV)**	**Log D [c]**
TC	9	190.2 ± 23.2	0.34	0.98 ± 0.5	2.1 ± 0.16 (2.6 ± 0.12*)
DDEA	7.6	59.3 ± 10.2	0.28	-9.19 ± 0.8	0.58 ± 0.06 (0.8 ± 0.04*)
ACV	5	130.9 ± 17	0.46	-0.62 ± 0.3	-1.65 ± 0.3 (-1.48 ± 0.7*)

Table S2. The hydrodynamic diameter of dextran (0.5 % w/v) in PBS, pH 7.4 and PBS, pH 5 at 24° C and 32 ^{o}C (n=3-5, 3 measurements per sample).

**Hydrodynamic diameter (nm)**				
**Vehicle**	**PBS, pH 7.4**		**PBS, pH 5**	
**Temperature (^{o}C)**	**24 ^{o}C**	**32 ^{o}C**	**24 ^{o}C**	**32 ^{o}C**
12 kDa dextran	11.7 ± 2.0	11.6 ± 0.9*	11.8 ± 3.5	12.2 ± 1.2*
80 kDa dextran	17.0 ± 1.1	18.0 ± 1.3**	17.9 ± 2.4	20.9 ± 1.8
150 kDa dextran	23.9 ± 2.9	21.6 ± 2.2	22.5 ± 1.5	23.3 ± 2.5

Expanded Method Details

Ex vivo skin studies

Full-thickness porcine skin was used in the ex vivo experiments as the appendages have been shown to remain open during ex vivo permeation studies rather than close like human skin. Adult pig ears were obtained from a local abattoir. The ears were removed from the carcass after hair removal. Any ears with visible skin damage were discarded. The ears were cleaned with water, the residual water on the skin surface was immediately removed by blotting with a tissue, visible residual hairs were trimmed carefully, and the ears were stored at -20 °C. Freezing has been shown not to compromise the permeability of porcine skin [1]. When required for the experiments, the porcine skin was thawed, and the subcutaneous fat was carefully removed using a scalpel. The skin was cut into 3.2 cm diameter circles and mounted with the SC facing the donor compartment in the Franz diffusion cell (University of Southampton, UK). The receptor phase consisted of a wetted sponge placed underneath the skin, ensuring direct contact was maintained with the dermal tissue. The hypobaric chamber was attached to the base and then the mounted cells were placed on a submersible stirring plate in a pre-heated water bath (Grant Instruments, Cambridge, UK) set at 37 ˚C, to obtain a temperature of 32 ˚C at the skin surface [2]. Five diffusion cells were used for each experiment. The studies were initiated by the application of an infinite dose of each in-house formulated gel (1 g) or by the application of either the purified or commercial FITC-dextran solution (1 ml, 500 mg/ml in PBS, pH 7.4) to the apical surface of the porcine skin. The studies were conducted for the duration of 24 h under atmospheric pressure (1010 mbar) or hypobaric pressure (500 mbar) applied for the first hour. The samples were maintained at room temperature until a quantitative determination by HPLC analysis (for diclofenac diethylamine, tetracaine, and acyclovir) and by Fluorescent spectroscopy (for the FITC-dextran 10 kDa, 70 kDa, and 150 kDa). Drug recovery testing in spent receiver fluid indicated that matrix interference with either assay was negligible (data not shown) and drug degradation was not detected using the Sephadex column for the entire duration of the experimental period.

At the end of the in vitro skin permeation studies, the Franz diffusion cells were dismantled, the skin was removed, wiped with water, and two tape strips were removed to ensure the removal of any residual formulation. The SC of the skin was removed by tape stripping (ca. 20 strips until the skin was translucent) using adhesive tape (Scotch 845 book tape, 3M, Bracknell, UK) as reported by Primo [3] (the first two strips were considered as part of the applied formulation. The remaining tape strips were applied sequentially by pressing the adhesive tape onto the skin using a roller to stretch the skin surface. Once the strips were removed, they were collected together, weighed and the drugs were extracted from them. Tetracaine and diclofenac diethylamine were extracted from the adhesive tape by immersing it in a solution of 90% methanol and 10% acetate buffer (0.1 M at pH 4) for 24 h. Acyclovir was extracted from the tape strips using a solution consisting of 95% hydrochloric acid (0.1 M pH 3) and 5% methanol. The extraction solutions containing the drug were dried down and the residue was reconstituted in acetate buffer (0.1 M) at pH 7.4 for diclofenac diethylamine, pH 5 for aciclovir, and at pH 9 for tetracaine and analyzed by HPLC. Once the *SC* had been removed, the epidermis was separated from the dermis using a scalpel as reported by Argenta [4]. Both the epidermis and dermis were homogenized (Ultra Turrax, Fisher Scientific, Leicester, UK) in the drug extraction solutions (compositions are mentioned previously) and the mixtures were incubated for 24 h at room temperature. The drug extract samples were then centrifuged at 17000 rpm (Biofuge, Heraeus, Germany) for 15 min, the resultant supernatant was evaporated. The drug residue that remained after evaporation was reconstituted in acetate buffer (0.1 M) at pH 7.4 for diclofenac diethylamine, pH 5 for acyclovir, and at pH 9 for tetracaine and analyzed by HPLC. To assess the extraction recovery efficacy, skin samples were spiked with a known amount of each drug, and the extraction procedure was conducted as previously described. The extraction recovery was measured by comparing the amount of each drug added and extracted. The extraction recovery of each drug from the receptor compartment was found to be 95.3 ± 2.5%, 93.4 ± 1.2%, and 96.1 ± 2.9% for tetracaine, diclofenac diethylamine, and acyclovir, respectively. Whilst the recovery extraction at 24 h period from the tape strips and skin tissue was found to be 96.16 ± 1.3% and 93.2 ± 5.2%, 94.4 ± 3.3% and 91.9 ± 7.2%, 95.3 ± 3.9%, and 95.8 ± 9.1% (n = 5) for tetracaine, diclofenac diethylamine, and acyclovir, respectively. Drug extraction was within the 100 ± 15% recovery rates, which was in line with published regulatory guidelines [5]. The effect of hypobaric pressure-controlled skin stretching upon drug cutaneous tissue deposition was represented by an enhancement ratio (ER) which was calculated as per the dextran studies.

[1] Weber L. The penetration of 2,3,7,8-tetrachlorodibenzo-p-dioxin into viable and non-viable porcine skin in vitro. Toxicology. 1993. 84:125-140.
[2] Maddock WG, Coller FA. The role of the extremities in the dissipation of heat. Am J Phys.1993. 106. 589-596.
[3] Primo F, Rodrigues M, Simioni A, Bentley M, Morais P, Tedesco A. In vitro studies of cutaneous retention of magnetic nanoemulsion loaded with zinc phthalocyanine for synergic use in skin cancer treatment. J Mag Magn Mat. 2008. 320: 211-214.
[3] Surber C, Schwarb FP, Smith, EW. Tape-stripping technique. J Tox Cut. Occ Tox. 2001. 20: 461–474.
[4] Argenta DF, Mattos, CB, Misturini FD, Koester LS, Bassani VL, Simões CMO, Teixeira HF. Factorial design applied to the optimization of lipid composition of topical antiherpetic nanoemulsions containing isoflavone genistein. Int J Nanomed. 2014. 9: 4737–4747.
[5] Health and consumer protection directorate-general. Basic criteria for in vitro assessment of dermal absorption of cosmetic ingredients. European commission (2006), SCCP/0970/06.
